# Assessment of Embedded Conjugated Polymer Sensor Arrays for Potential Load Transmission Measurement in Orthopaedic Implants

**DOI:** 10.3390/s17122768

**Published:** 2017-11-29

**Authors:** Carolina Micolini, Frederick Benjamin Holness, James A. Johnson, Aaron David Price

**Affiliations:** 1 Organic Mechatronics and Smart Materials Laboratory Mechanical and Materials Engineering, University of Western Ontario, London, ON N6A 5B9, Canada; cmicolin@uwo.ca (C.M.); fholness@uwo.ca (F.B.H.); 2 Lawson Health Research Institute, University of Western Ontario, London, ON N6C 2R5, Canada; jim.johnson@sjhc.london.on.ca

**Keywords:** intrinsically conductive polymers, piezoresistance, polyaniline, sensing array, orthopaedic joint implants, reverse total shoulder arthroplasty, conjugated polymers

## Abstract

Load transfer through orthopaedic joint implants is poorly understood. The longer-term outcomes of these implants are just starting to be studied, making it imperative to monitor contact loads across the entire joint implant interface to elucidate the force transmission and distribution mechanisms exhibited by these implants in service. This study proposes and demonstrates the design, implementation, and characterization of a 3D-printed smart polymer sensor array using conductive polyaniline (PANI) structures embedded within a polymeric parent phase. The piezoresistive characteristics of PANI were investigated to characterize the sensing behaviour inherent to these embedded pressure sensor arrays, including the experimental determination of the stable response of PANI to continuous loading, stability throughout the course of loading and unloading cycles, and finally sensor repeatability and linearity in response to incremental loading cycles. This specially developed multi-material additive manufacturing process for PANI is shown be an attractive approach for the fabrication of implant components having embedded smart-polymer sensors, which could ultimately be employed for the measurement and analysis of joint loads in orthopaedic implants for in vitro testing.

## 1. Introduction

Polyaniline (PANI) is an established conductive organic polymer that has been thoroughly investigated. In several studies, it has been established that PANI has a negative gauge factor (GF) [1,2]. The gauge factor is the ratio of relative change in electrical resistance *R*, as a function of the mechanical strain, and it characterizes the sensitivity of piezoresistive materials [3]. This feature, in addition to its low-cost, ease of synthesis, good environmental stability, and in-vitro and in-vivo biocompatibility [4], suggests that PANI is a plausible candidate for the fabrication of piezoresistive sensors [5,6,7,8,9]. However, the use of this polymer for the manufacture of sensors based on its piezoresistive properties warrants further investigation [10]. There have been many approaches to study PANI as a sensing element: Barra et al. [11] investigated the behaviour of various thermoplastic elastomer/PANI blends under compressive stress to assess their electromechanical properties, Pereira et al. [2] examined the piezoresistive effect of spin-coated PANI thin films, Castillo-Castro et al. [10] studied the piezoresistive response to compressive loads on composites that featured hydrochloric PANI (PANI-Cl) as an electrically conducting filler, DellaPina et al. [3] compared electromechanical properties in response to compression for PANI pellets obtained following two different approaches: a traditional method and a green method. Despite the variation in PANI synthesis methods, blending, and experimental testing, the piezoresistive response in all cases reinforces that PANI is an interesting candidate for pressure sensor applications. Bao et al. [6] report that the conductivity of PANI may vary depending on its composition, the synthesis method, and the treatment it receives. Our approach uses a special technique for the 3D-printing of PANI, which could assure a reproducible printing pattern among the sensing elements. In a previous study, the PANI utilized for this investigation has been established to have a GF that ranges from 6.42 to 5.09 [12].

An exciting application of the aforementioned technology is the use of embedded transducers within orthopaedic implants, as load transfer through these devices is poorly understood. One example is the reconstruction of the shoulder which is an operation becoming very popular and in high demand with the “baby-boomer” population. Total reverse shoulder arthroplasty (RSTA) is, in particular, a relatively new procedure that has shown promising short-term results for the treatment of glenohumeral arthritis and massive rotator cuff tears, and as a salvage procedure following failure of unconstrained anatomical total shoulder arthroplasty [13,14,15]. The glenohumeral joint is a ball and socket type joint formed between the articulation of the rounded humeral head and the rounded socket (glenoid fossa) of the scapula. It is a muscle-dependent joint as it lacks strong ligaments. In a reverse total shoulder replacement the articulation is reversed: the articulating head, a spherical cobalt chromium glenoid component, is fixed to the scapula and the socket, a convex ultra-high molecular weight polyethylene (UHMWPE) humeral component, is fixed to the upper end of the humerus. The reverse shoulder replacement is relatively new, therefore the longer-term outcomes of this implant are just starting to be understood. It is known that failure rates can be as much as four times higher with respect to anatomical shoulder replacements, but nonetheless this approach presents great benefits for cases in which the latter cannot be used [16]. The joint reaction force reaches the approximate equivalence of half of a body weight (350N for an average adult male) [15,17,18] for the reversed prosthesis. Additionally, it has been estimated that the average of the maximum contact stresses, while applying a constant load of 400N, ranges from 2.28MPa to 3.28MPa found for a commercially available 38mm humeral cup ($155^{\circ }$ and $145^{\circ }$ Neck-Shaft angle, correspondingly), and from 2.1MPa to 4.48MPa in a commercially available 42mm humeral cup ($155^{\circ }$ and $145^{\circ }$ Neck-Shaft angle, correspondingly) [19].

**Figure 1:**
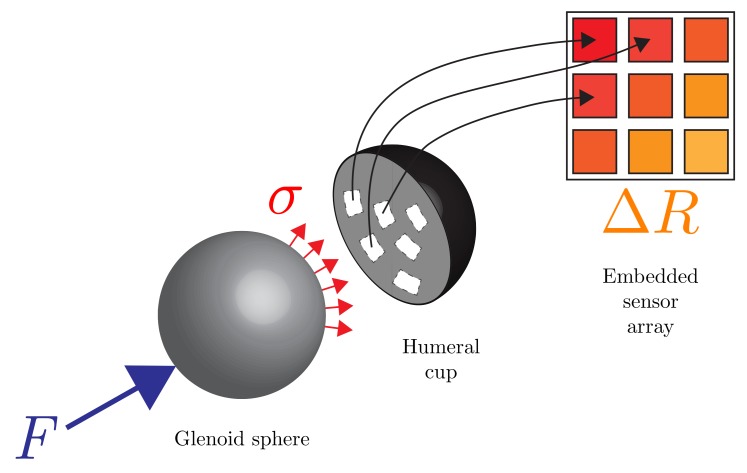
Conceptual representation of an orthopaedic implant with an embedded smart polymer sensor network.

This work ultimately aims to implement the design of a 3D-printed polymer sensor using conductive PANI structures as embedded smart-polymer sensors within the humeral component of the prosthesis used in RTSA procedures. The motivation for this design is to elucidate the stress distribution on the humeral cup and identify possible critical wear patterns in the humeral component. Previous efforts to measure contact points have been predominantly limited to strain gauges to measure contact loads at discrete points along the implant interface. It is imperative to monitor contact loads across the entire area, wherein the measurement of loads at discrete points on (or within) the implant is of particular interest since, while certain joint forces measured using the aforementioned techniques do not seem significant in terms of body weight, if these loads are concentrated on a single point they can result in wear and plastic deformation of the UHMWPE component [20]. The sensing technique proposed herein will lead to an improved understanding of force transmission mechanisms encountered by these implants in service leading to better designs, and ultimately, to extended implant lifespans [18,21,22,23,24]. Unlike when strain gauges are employed, incorporation of polymer-based sensors enables the stiffness characteristics of the implant interface to be maintained, which leads to more accurate measurement of the true internal loads. This study presents the characterization of PANI as a sensing element for internal forces in joint implants as conceptualized in Figure 1. In this regard, a 3D-printed planar sensor was realized having a 3×3 array of PANI rectangular prisms embedded within a flexible polymeric substrate.

## 2. Materials and Methods

### 2.1. Design and Fabrication

**Figure 2:**
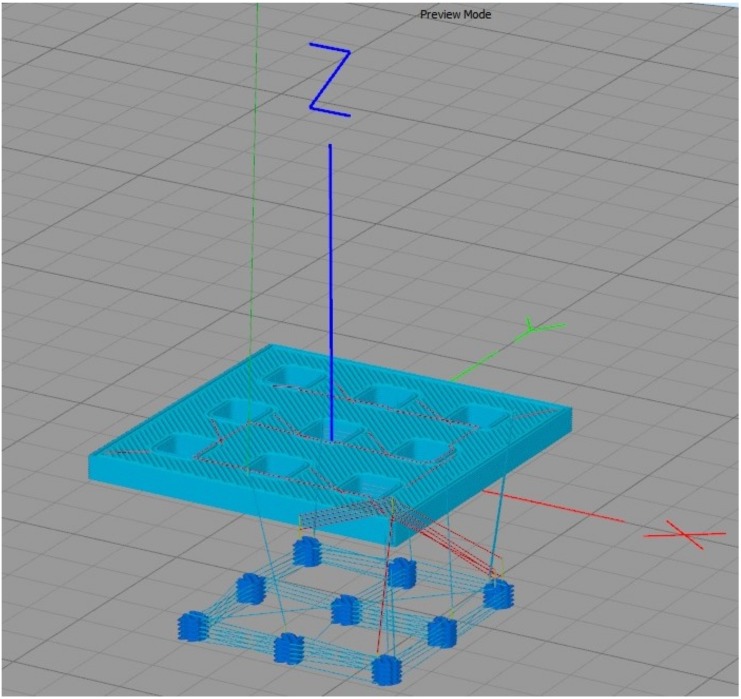
Pre-visualization generated by Simplify3D of the printing pattern, showing print lines and fill pattern.

The piezoresistive property of PANI was harnessed as a pressure sensor for the 3D-printed part. The model of the sensor was created using SolidWorks 2016 R2016a (Dassault Systèmes, Vélizy-Villacoublay, France) in a 3×3 array of PANI rectangular prisms embedded in a flexible polymeric substrate. This pattern was created to demonstrate that it is possible to distinguish between different pressures in various areas, monitoring across the whole surface. The model of the flexible polymeric substrate was 3D-printed using 1.75mm Cheetah filament, a thermoplastic elastomer (TPE) filament in the shore hardness 95A (Ninjatek, Manheim, PA, USA) and PANI doped by the use of dodecylbenzene sulfonic acid (DBSA) prepared according to the protocols described by Holness et al. [25]. To this end, a specialized multi-material 3D printing technique has been developed at the Organic Mechatronics and Smart Materials Laboratory for direct-ink writing processes using a modified fused filament fabrication delta robot equipped with an integrated polymer paste extruder, which has been discussed in detail elsewhere [12,25,26]. In accordance with this technique, PANI was dispensed using a 22 Gauge stainless steel tip (Nordson EFD, East Providence, RI, USA), in a rectilinear $\left(\pm 45^{\circ }\right)$ fill pattern, and without a perimeter wall. The flexible polymeric substrate includes a removable base where the PANI is deposited to affix the part onto the build plate during printing, as well as 3 solid layers in the bottom and top and 2 perimeter outlines. It was printed with rectilinear $\left(\pm 45^{\circ }\right)$ infill configured to 15%, to ensure that the TPE was deformed along with the PANI, and to ensure that it had sufficient structure to fully envelope the PANI. Figure 2 shows a pre-visualization generated by Simplify3D (Simplify3D, Cincinnati, OH, USA) of the printing pattern, showing the print trajectory and fill pattern.

The dimensions of the sensor were 30mm×30mm×2.5mm. This dimension represents the approximate area of the humeral cup of an RTSA. The size of each PANI rectangular prism, the shape prescribed to facilitate their 3D printing simultaneously with the TPE, was 3.5mm×3.5mm×2.5mm. PANI elements were printed leaving a gap of 0.5mm between the PANI and the TPE to allow for deformation and to avoid the overlap of the two materials during printing. The distance between the centres of each element was 9mm. The unwired-sensor was weighed before the thermal treatment at $165{\;}^{\circ }C$ for 5min on a covered hot plate [10] and subsequently wired with electrical connections. Two methods of connection were tested to join the wires with the PANI elements: the use of conductive carbon tape and the use of silver epoxy as suggested by Blythe [27], who indicates that the uncertain contact resistances between the electrodes and the specimen can be reduced using silver paint. The accuracy by means of Root Mean Square Error (RMSE) was calculated for both methods at all load levels, with the average error ranging from 3.72–5.38% in the case of silver epoxy interconnects, while accuracy in the carbon tape samples ranged from 17.7–74.94%. The extreme values encountered in the latter case indicate an unstable connection between the wires and the PANI, which suggest discarding the conductive carbon tape approach in favour of silver epoxy (MG Chemicals, Surrey, BC, Canada) as the preferred means of connection.

### 2.2. Signal Acquisition and Processing

The piezoresistive effect consists of a change of the electrical resistivity of a semiconductor when mechanical strain is applied. A through-thickness sensing mode was employed to measure this change in resistance [9]. Ten voltages were acquired using a 32-input compact DAQ NI 9205, mounted in a NI 9174 chassis (National Instruments, Austin, TX, USA). Nine of these analog inputs were used to measure the voltages from the top surface of each PANI element, and one analog input was used to measure the applied voltage (nominal value of 2V) input to the two-point probe used to measure the sensor resistance. The connection made between all the elements on the underside was connected to the ground in the circuit and in the COM channel of the DAQ NI 9205. A custom-made MATLAB (MathWorks, Natick, MA, USA) software interface was developed to analyze the voltage signal and to calculate the resistance. A voltage-divider method was used to measure the resistivity according to the following equation:

(1) $$R=\frac{R_{S}}{(\frac{V_{\mathrm{in}}}{V_{R}})-1},$$

where *R* is the variable PANI resistance measurand, $R_{S}$ is the shunt resistor of 1000Ω±1% tolerance, in series with *R*, $V_{\mathrm{in}}$ corresponds to the input voltage to the whole circuit, including *R* and $R_{S}$, and $V_{R}$ is the voltage drop across *R*. The input voltage can be defined by the user, in this case, $V_{\mathrm{in}}=2\;V$. A filter was employed to introduce 60Hz noise rejection while decreasing noise rejection at other frequencies. All negative resistivity was remapped to null resistivity. Equation (1) was used to measure *R* in each PANI element during each individual pressure load. After the range of the pressure was acquired, curve fits were applied to each data set (there are nine sets of data points, one for each PANI element). All tests were performed under a nominal excitation voltage of 2V.

### 2.3. Calibration Apparatus

**Figure 3:**
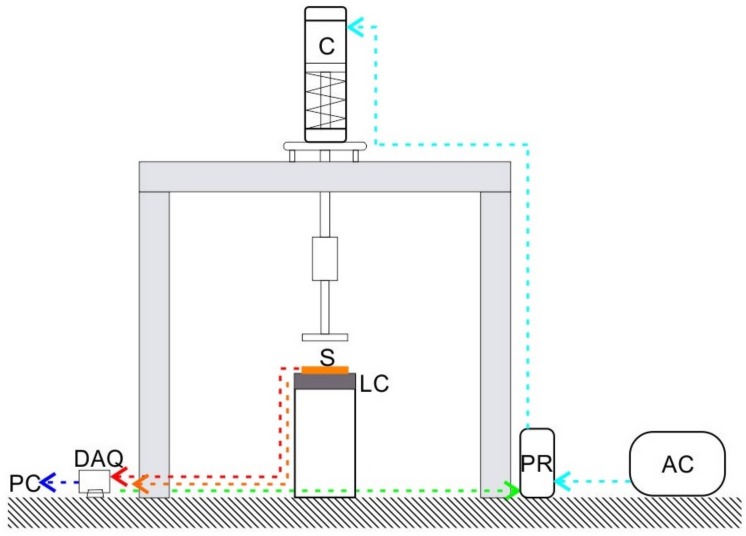
Calibration apparatus consisting of Air Cylinder (C), Pressure Regulator (PR), Air Compressor (AC), Data Acquisition System (DAQ), Load Cell (LC), and Personal Computer (PC). A Sensor (S) is illustrated to demonstrate the placement at the time of characterization.

Compression tests were carried out at room temperature using a specially designed calibration apparatus as depicted in Figure 3. This apparatus consists of a pneumatic cylinder (Bimba, University Park, IL, USA) in which the air flow was controlled by a QPV1 electronic pressure regulator (Equilibar, Fletcher, NC, USA) connected to an air compressor. A flat surface of 30mm×30mm was used to apply uniform pressure to the sensor. The electronic pressure regulator was driven by a NI 9263 (National Instruments, USA) on an NI 9174 chassis (National Instruments, USA). The applied load was corroborated by a LCAE 35 KG load cell (Omega, Norwalk, CT, USA). The pressure regulator was driven by a data acquisition system NI 9263, and the load cell output was captured using a DAQ 9205 via LabVIEW interface.

The software acquired the resistance measured in each PANI element during each individual pressure load. After the range of the resistance values was acquired, linear curve fits were applied to each grouping of data sets (there are nine sets of data points, one for each PANI element). A graphical user interface was created that relates the sensed resistance measurements to the calibrated applied pressure such that the sensor array can be harnessed to monitor unspecified pressure loads.

### 2.4. Sensor Array Characterization

After the sensor was printed, thermally treated, and instrumented, a suite of piezoresistive characterization experiments were undertaken utilizing the specially designed calibration apparatus previously described via a series of six individual experiments.

#### 2.4.1. Stability

The baseline resistance of each element without any applied load was first established, and then measured under a constant load for a given period of time. The resistance of the elements was measured with no applied load for the first 160s, in a total of 12 measurements. Afterward, a 6.78N load was applied and the elements were left to stabilize for 35s, after that time the first measurement of the resistance under load was taken, with a total of 41 measurements under load taken in a 1966s period.

#### 2.4.2. Cycle Loading

Loading/unloading cycles of the elements were carried out, applying the same load in each cycle. Loaded measurements were taken using a 27.58kPa input for the air cylinder, corresponding to a 5% input of the 551.58kPa supplied by the air compressor of the calibration apparatus, which applies a total load of 6.78N onto the sensor. The initial measurement was taken with no applied load $\left(R_{0}\right)$, the sensor was then loaded for 30s, in order to allow the PANI to stabilize at the new load level of 8.5N [3], subsequently the loaded measurement $\left(R_{5\%}\right)$ was taken. After this measurement was taken, the sensor was left unloaded for 30s before a new cycle. This process was repeated for 40cycles, with a total of 80 resistance measurements. Plots were obtained by introducing the following relations:

(2) $$\begin{array}{c} Point_{0,n}=\frac{R_{0,n}-R_{0,n-1}}{R_{0,n-1}}, \end{array}$$

(3) $$\begin{array}{c} Point_{5\%,n}=\frac{R_{5\%,n}-R_{0,n}}{R_{0,n}}, \end{array}$$

where $R_{0}$ corresponds to the value for the unloaded-resistance measured, and $R_{5\%}$ corresponds to the value for the loaded-resistance measured.

#### 2.4.3. Incremental Loading

Two distinct loading approaches were employed to characterize the sensor performance: continuous incremental (staircase) loading, and zero-break (also known as zero-max) loading. In the former case, loads were applied from 0N to 50.84N in 3.4N increments, and left to stabilize for 60s. In the latter case, loads were applied incrementally from 20.34N to 84.73N in 3.4N increments, but between load increments the load is fully removed for 60s. After each increment, the elements were left to stabilize for 60s prior to measurement. For each measurement, the fractional change in resistance ($\Delta R/R_{0}$) is determined. ΔR is the difference between the resistances measured under load and the unloaded reference state ($R_{0}$), and normalized by the unloaded resistance, $R_{0}$.

#### 2.4.4. Loading/Unloading Cycle

A loading/unloading cycle carrying the load from 0N–50.84N, in steps of 8.5N was performed. Then the load was decremented in steps of 8.5N, carrying the load from 50.84N–0N. During the loading cycle, the elements were subjected to loading for 60s before taking the resistance measurement, after taking the measurement the load was increased again. During the unloading cycle, the load was decremented, and the elements were subjected to the new load for 180s before taking the new measurement. The first measurement of the unloaded resistance was considered as 100% of the resistance value, and the remaining resistances were calculated as a percentage of this baseline.

#### 2.4.5. Repeatability

Three loading/unloading cycles were performed, letting the elements to rest in between each cycle for a minimum of one hour. To enable facile interpretation of the sensor output, only the subset of data collected in the load cycles from 5% up to 30% of the 551.58kPa input for the pneumatic cylinder are presented.

#### 2.4.6. Accuracy

To calculate accuracy, the root mean square (RMS) of the error between the measurements taken in each one of the three loading/unloading cycles compared to the average of the measurements obtained in all the cycles was calculated, using the following formula:

(4) $$RMSE=\sqrt{\frac{1}{n}\sum_{i=1}^{n}{\left(\frac{R_{n}-\bar{R}}{\bar{R}}\times 100\right)}^{2}}.$$

## 3. Results

As the ultimate goal of this project is to realize fully-instrumented orthopaedic implants using embedded conductive polymer arrays in complex geometric arrangements, this first study aims to assess the baseline performance of planar 3D-printed PANI-based piezoresistive sensor arrays. To this end, the piezoresistive characteristics of PANI sensors prepared via this approach were experimentally characterized as follows.

### 3.1. Design and Fabrication

**Figure 4:**
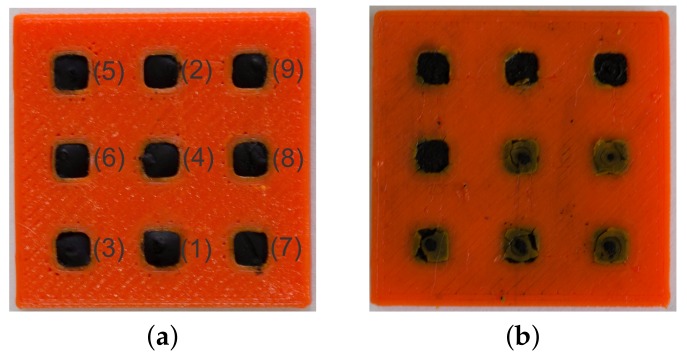
3D-printed sensor array without the wires (**a**) Front view, with element array labels and (**b**) Back View - bottom elements showing the base used to contain the deposited PANI.

**Figure 5:**
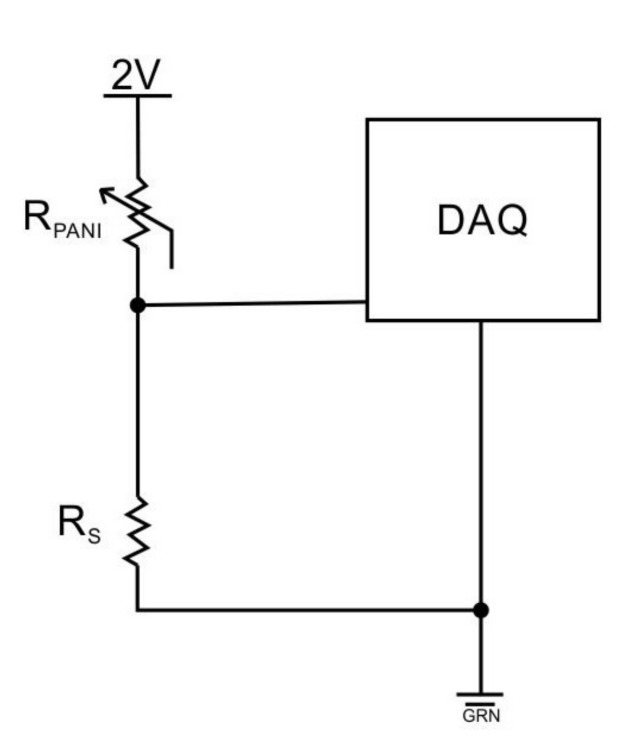
Schematic connection for one of the sensor elements consisting of a PANI element ($R_{\mathrm{PANI}}$), shunt resistor ($R_{S}$), and data acquisition system (DAQ) with a 2V excitation.

**Figure 6:**
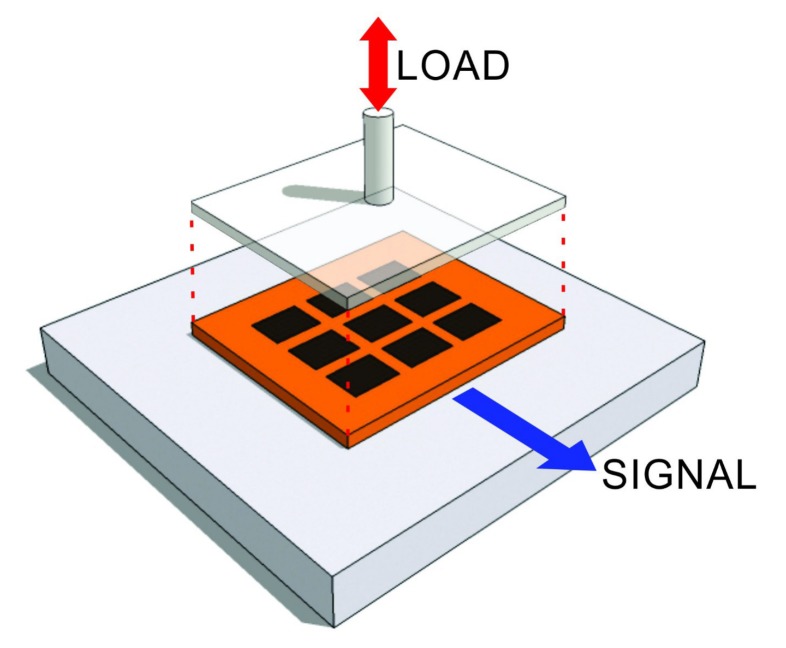
Scheme of the sensor configuration for piezoresistive characterization.

As shown in Figure 4, the fabricated sensor consists of a 3×3 array of individually addressable PANI rectangular prisms embedded in a flexible polymeric substrate. A schematic of the electrical connection for each individual element is illustrated in Figure 5. Figure 6 depicts the sensor configuration during all tests of piezoresistive characterization. The unwired-sensor was weighed before the thermal treatment of $165{\;}^{\circ }C$ for 5min on a covered hot plate, its initial weight was 1.8980g. It was weighed again immediately after, and the final weight was 1.8575g. The 0.0405g difference is due to the loss of moisture by evaporation [11].

### 3.2. Sensor Array Characterization

#### 3.2.1. Stability

**Figure 7:**
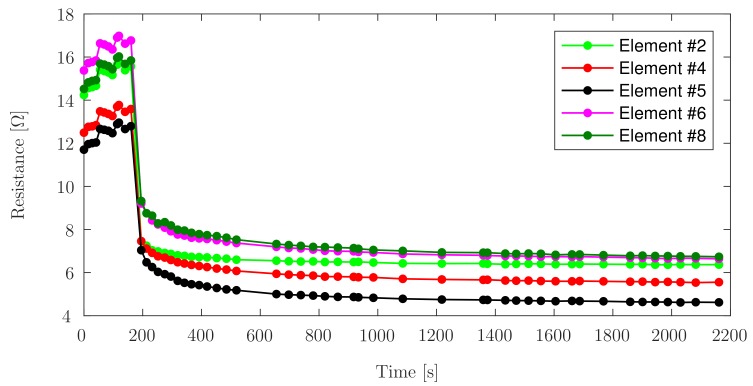
Change in resistance under no load and continuous load, for each PANI element.

In this test set, Element ^#^9 was discarded due to wire connection problems. Element ^#^1 and Element ^#^7 exhibit an abrupt drop in measurement for the same reason. When the sensor was unloaded, the wires did not retain sufficient electrical contact, which corresponds to the high resistance values observed. Once the load is applied, the force generated by the pneumatic cylinder is sufficient to ensure good electrical contact. Figure 7 shows the measurements of Elements ^#^2, ^#^4, ^#^5, ^#^6 and ^#^8, and depicts a stable response even without load.

The initial abrupt drop during the first seconds of the load can be attributed to the delay of the pneumatic piston to reach the desired load value. For all elements, the value of the resistance reaches its final stabilization around the 7th minute of continuous load application. If the first loaded measurement of resistance for each element is considered as 100% of the value for that series, the percentage dropped to a maximum of 55.7% of its initial value for Element ^#^1 and a minimum of 85.45% for Element ^#^2, both are the final measurements taken, with the final values of the other elements ranging between these values.

#### 3.2.2. Cycle Loading

Two relations have been employed to characterize cyclic loading behaviours: Equation (2) shows the difference between the measurements of unloaded-resistance versus the previous unloaded-resistance, while Equation (3) indicates the difference between the measurements of the loaded-resistance versus the value of the unloaded-resistance previous to the loading of the elements. The disturbance observed in both graphs in cycles 37 and 38 for Element ^#^5 are attributed to an overload of 170N for this element after cycle 36.

#### 3.2.3. Incremental Continuous Loading

**Figure 8:**
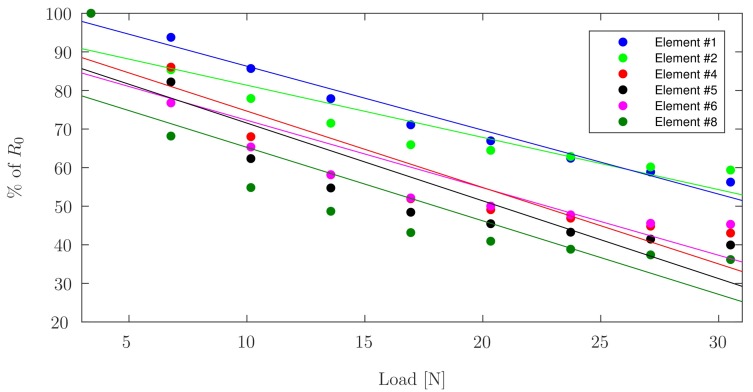
Linear fits for the change in resistance as a function of $R_{0}$ during incremental loading, for each PANI element.

The value of the resistance at 3.4N of applied force ($R_{0}$) is considered to be 100%, while all other values are represented as a percentage of $R_{0}$. Figure 8 indicates the change in resistance for each PANI element during incremental continuous loading up to 30.5N, above which the response tends to plateau.

#### 3.2.4. Incremental Loading: Zero-Breaks

**Figure 9:**
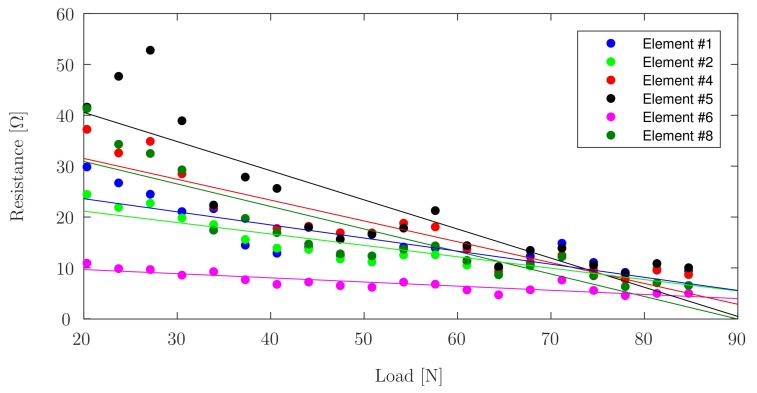
Linear fits for the change in resistance as a function of $R_{0}$ during incremental loading-zero-breaks, for each PANI element.

Element ^#^3 and Element ^#^9 were omitted from Figure 9 as the former did not exhibit significant change in resistance under load, while the latter exhibited erratic behaviour. Both outliers are attributed to faulty electrical connectivity.

#### 3.2.5. Loading/Unloading Cycle

**Figure 10:**
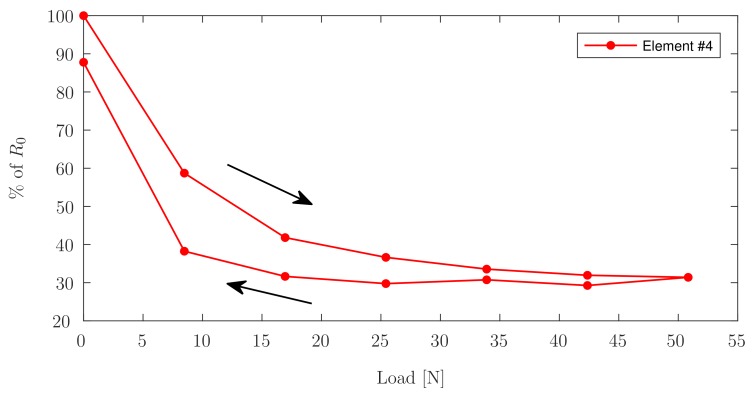
Relative change in resistance on Element ^#^4 during the first loading/unloading cycle.

As all elements exhibit similar behaviour, Element ^#^4 is shown as a representative element to visualize the graph without interference. Figure 10 shows the values measured for the relative change in resistance of Element ^#^4 during the first loading/unloading cycle.

#### 3.2.6. Repeatability

**Figure 11:**
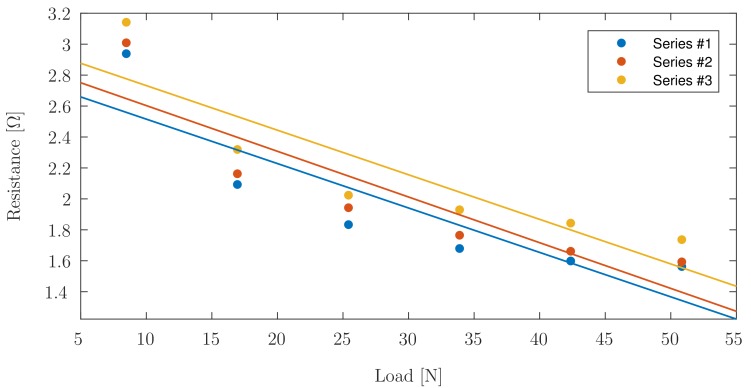
Change in resistance on Element ^#^4 during the loading portion of the three repetitions of loading/unloading cycles as a function of load.

Figure 11 suggests that the dynamics of the response are consistent across all three cycles. Table 1 indicates the $R^{2}$ values for the linear fitting; only values for elements with functional connectivity have been included.

#### 3.2.7. Accuracy

The maximum value of RMSE is found for Element ^#^6, with a value of 7.97%, while the minimum value is found for Element ^#^5, having an RMSE of 0.66%. All other elements have RMSE values in between, as reported in Table 2. Across all load levels, the elements show an average error ranging from 3.72–5.38%.

## 4. Discussion

**Figure 12:**
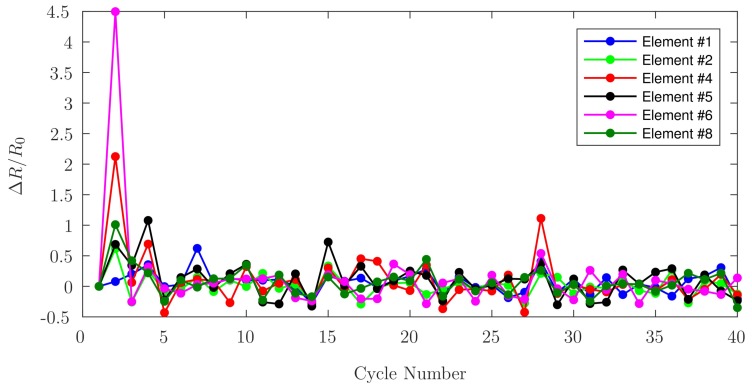
Fractional change in unloaded-resistance as a function of cyclic loading for each PANI element.

**Figure 13:**
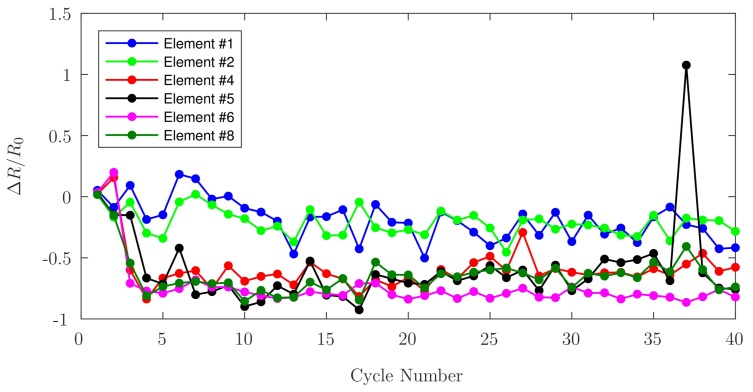
Fractional change in loaded-resistance as a function of cyclic loading for each PANI element.

In this study, a series of experiments to characterize and evaluate printed conductive PANI as a potential candidate for the fabrication of implant components having embedded smart-polymer sensors was executed. The response of PANI to the cyclical application of loads, its repeatable response to incremental compressive loading, the stability of its response to a continuous load, and the accuracy of these measurements, were used to corroborate the feasibility of this material as a potential sensor. Due to the 3D printing capabilities currently available, it is not possible to ensure an identical PANI deposition for each sensing element, at the same time the different amounts of silver epoxy used for the wire connections added to the lengths of these wires translate to different resistances values for each sensing element. The wires were positioned to ensure similar heights among them, but for the aforementioned reasons, they may have had slight height differences which may have produced an uneven loading behavior. After all the test cycles were performed, and upon close inspection of the elements, the wires were not notably embedded in the PANI. After the cyclical application of loads, two separate relations have been used to infer trends: Equation (2), used for Figure 12, shows that the difference between the measurements of unloaded-resistance versus the previous unloaded-resistance tends to zero. This behaviour is consistent with the resistance returning to its pre-load value when no load is applied. This behaviour is consistent with the observations reported by Barra et al. [11], who attribute re-organization of conduction pathways and elastic deformation of the matrix as the mechanisms for relative conductivity returning to its previous value upon unloading. Figure 13 shows the difference between the measurements of the loaded-resistance versus the baseline value of the unloaded-resistance. During the cyclic loading, it was observed that the measured resistance values for each element increase with each cycle in accordance with the observations made by Falletta et al. [8], who suggest that irregular behaviour may be attributed to initial mechanical instabilities within the polymers. Both figures indicate that the fractional change in PANI electrical resistance becomes more stable after 30cycles.

It can be seen in Figure 8, showing linear trend lines for the data acquired during incremental continuous loading, that all elements have an initial nonlinear region, followed by a linear region in which PANI is best suited as a sensing element. Both incremental continuous loading and incremental loading with zero-breaks confirm faulty behaviour for Elements ^#^3, ^#^7, and ^#^9. This phenomenon can be confirmed since Elements ^#^3 and ^#^7 do not show notable changes in resistance measured under load or without load, and Element ^#^9 shows erratic behaviour corresponding to connectivity problems. Figure 11 shows the linear fit for the data acquired during the three cycles of piezoresistive characterization on Element ^#^4, where the resistance is plotted as a function of the applied load. Linear curve fits were applied to each set of measurements. $R^{2}$ values were obtained as high as 0.8608 for the first cycle of Element ^#^1, with an average value of 0.7803, and the lowest value found for Element ^#^6 during the second loading/unloading cycle with a value of 0.6447. With the application of increasing loads, the resistivity of PANI decreased linearly. Prior studies have observed that the reduced interchain separation coupled with the enhanced interchain charge transport diminish the resistance of PANI while enduring compressive load [6,8,11]. This demonstrates that while the response of the material is reliable, their main limitation lies in the fragility of the connections between the PANI and the wire leads, which tend to loosen after a low number of loading cycles, but once a load is applied, the force generated is sufficient to ensure good electrical contact between the wires and the PANI elements. Other connection approaches including silver filled electrically conductive adhesives are currently being explored to improve these connections. Future efforts will focus on the development of a 3D-printed instrumented humeral cup, consistent of these PANI-based embedded sensors, as shown in Figure 1. In accordance with the region in which PANI was found to show a linear behaviour and aforementioned expected loads in a RSTA, a greater quantity of sensor elements will be used on the surface of the humeral cup to measure the joint load. The mechanical load frame employed to verify the load-voltage relationships in this investigation is subject to inaccuracies, therefore in the future a dedicated load frame will be employed. Due to the geometry of the humeral component of these implants, it will be necessary to make modifications in the calibration apparatus in order to apply a force perpendicular to each PANI element at the time of the calibration. Furthermore, it will be necessary to adjust the custom-made MATLAB (MathWorks, USA) software interface to calibrate each PANI element individually and not simultaneously as is currently done for the planar sensor.

## 5. Conclusions

The design, fabrication, characterization, signal acquisition and processing of a 3D-printed polymer sensor with a 3×3 conductive PANI structure array was presented in this investigation. The piezoresistive characterization of PANI elements embedded within a sensor array, in addition to the calibration apparatus and associated techniques, were conveyed. The fractional change in resistance upon continuous loading, cyclical loading and unloading, and incremental loading, in addition to the repeatability and accuracy of the loading/unloading cycles were described. As the developed PANI sensor arrays exhibited linear outputs between the fractional changes in resistance during incrementally applied loads, together with a stable response, this specially developed multi-material additive manufacturing process for PANI represents an attractive candidate for the manufacture of implant components having embedded smart-polymer sensor arrays. The next phase of this project will build upon this work to fabricate a concave sensor that mimics the humeral cup geometry of a reverse total shoulder arthroplasty implant.

## Figures and Tables

**Table 1:** $R^{2}$ values for the linear fitting.

	Element ^#^1	Element ^#^2	Element ^#^4	Element ^#^5	Element ^#^6	Element ^#^8
Series 1	0.81	0.85	0.76	0.79	0.78	0.81
Series 2	0.86	0.68	0.79	0.80	0.64	0.81
Series 3	0.84	0.68	0.78	0.78	0.71	0.82

**Table 2:** RMS of the error between the measurements taken in each one of the three loading/unloading cycles compared with respect to the average of the measurements obtained in all the cycles.

Load [N]	Element ^#^1	Element ^#^2	Element ^#^4	Element ^#^5	Element ^#^6	Element ^#^8
8.47	5.47%	7.19%	2.78%	0.66%	5.90%	4.88%
16.95	6.27%	5.19%	4.32%	1.99%	7.97%	5.00%
25.42	4.99%	4.49%	4.03%	2.86%	1.65%	4.41%
33.89	6.01%	5.49%	5.80%	5.24%	4.40%	3.35%
42.36	4.53%	2.71%	6.10%	6.34%	4.42%	2.98%
50.84	2.98%	7.28%	4.65%	5.21%	6.51%	4.47%
